# College Students’ ADHD Symptoms and Alcohol Response During Laboratory Self-Administration

**DOI:** 10.3390/ejihpe16070091

**Published:** 2026-06-29

**Authors:** Amy L. Stamates, Sabrina M. Todaro, Anna L. Sherman, Melissa C. Rothstein, Dahianna López, Lisa L. Weyandt

**Affiliations:** 1 Department of Psychology, University of Rhode Island, 142 Flagg Road, Chafee Hall, Kingston, RI 02881, USA; sabrinamtodaro@uri.edu (S.M.T.); anna_sherman@uri.edu (A.L.S.); mrothstein@uri.edu (M.C.R.); lisaweyandt@uri.edu (L.L.W.); 2 College of Nursing, Providence College, Providence, RI 02918, USA; dlopez6@providence.edu

**Keywords:** alcohol administration, subjective alcohol response, perceived intoxication, alcohol craving, college students, ADHD

## Abstract

Background: Alcohol use and attention-deficit/hyperactivity disorder (ADHD) are prevalent among college students. Individuals with ADHD are at increased risk for developing alcohol use disorder, but mechanisms contributing to this risk are unclear. Subjective alcohol response, or how one experiences the effects of alcohol use, is an important factor that contributes to alcohol consumption. Research suggests that individuals with ADHD, as compared to those without ADHD, may differentially experience the effects of alcohol. Consequently, the purpose of the present study was to examine the association between ADHD symptoms and subjective alcohol response (i.e., subjective effects of alcohol, perceived intoxication, and craving). Methods: Participants (*N* = 26; 38.5% male, 61.5% female) were college students who completed an in-person alcohol administration session where they received a 0.65 g/kg dose of alcohol. Breath alcohol concentrations and subjective effects of alcohol (feel, like, sedation, and stimulation), perceived intoxication (intoxication and willingness to drive), and craving were assessed at multiple time points throughout the session. ADHD symptoms were assessed at baseline. Results: Area under the curves (AUC) were generated for each participant and each subjective effect, perceived intoxication, and craving. Controlling for typical alcohol use, multiple regressions revealed that higher ADHD symptoms were associated with greater liking the drink, willingness to drive, and craving AUC scores. Conclusions: Individuals with higher ADHD symptoms may experience greater liking and craving during a drinking session. The present findings may also be useful to consider when tailoring intervention and prevention efforts to help reduce risk for college students who report elevated ADHD symptomology.

## 1. Introduction

Alcohol use (Substance Abuse and Mental Health Services Administration [SAMHSA], 2025) and attention-deficit/hyperactivity disorder (ADHD) are both prevalent among college-aged adults (Weyandt & DuPaul, 2008). Further, ADHD has been associated with greater experiences of alcohol-related consequences and alcohol use disorder (AUD; Falco et al., 2023; Glass & Flory, 2012; Luderer et al., 2020). Although a burgeoning literature suggests that higher ADHD symptoms (i.e., inattention and hyperactivity/impulsivity; American Psychiatric Association [APA], 2013) are associated with a greater risk of alcohol-related consequences (Blasé et al., 2009; Curry et al., 2019; Falco et al., 2023), mechanisms underlying this association are unclear. For example, there is conflicting evidence on the link between ADHD symptoms and level of alcohol use. Some studies suggest that ADHD symptoms may be related to greater alcohol use (Dattilo et al., 2013; Falco et al., 2023), while other studies suggest that the association between ADHD symptoms and alcohol use is nonsignificant (Glass & Flory, 2012; Mesman, 2015). Given the high comorbidity between ADHD and AUD (Kennedy et al., 2026), there is an urgent need to identify other mechanisms, beyond the level of alcohol use consumed, contributing to alcohol-related risk among young adults with ADHD symptoms.

Subjective alcohol response, or how one experiences the pharmacological effects of alcohol, has been associated with an increased risk of AUD (K. M. King et al., 2014; Schuckit et al., 2004; Trim et al., 2009). Aspects of subjective alcohol response may include factors such as how an individual “feels” the alcohol, “likes” the alcohol, and how stimulated or sedated an individual feels as a result of drinking alcohol (Fillmore & Rush, 2001; Martin et al., 1993). Higher alcohol craving, alcohol enjoyment, and greater stimulative effects have been shown to be related to greater alcohol consumption (A. C. King et al., 2011; A. King et al., 2021). The Differentiator Model supports this research, as this theory posits that greater subjective effects of alcohol (i.e., sedation and stimulation) may serve as cues that reinforce continuing and/or increasing alcohol use (Newlin & Thomson, 1990). Contrastingly, the Low-Level Response Model suggests that lower subjective effects of alcohol (i.e., feeling fewer sedative effects) may motivate greater alcohol use due to the absence of cues that typically signal to stop or slow the pace of drinking (Schuckit, 1994). Taken together, these models support that the subjective experience of alcohol is an important mechanism that contributes to alcohol consumption.

Scant research has examined the associations between ADHD symptoms and subjective effects of alcohol, perceived intoxication, and alcohol craving (Hendershot et al., 2015; Roberts et al., 2013; Weafer et al., 2008). For example, one study measuring ADHD symptoms and subjective alcohol response during acute intoxication in a sample of 19–21-year-olds found that higher ADHD symptoms were associated with increased stimulation (Hendershot et al., 2015). Other alcohol administration studies have suggested that individuals with ADHD may feel less intoxicated than those without ADHD following a high (0.65 g/kg) dose of alcohol (Weafer et al., 2008), especially as blood alcohol concentrations fall (Roberts et al., 2013). These results suggest that individuals with ADHD may not accurately evaluate their level of intoxication, which may potentially lead to a higher risk of consequences (e.g., driving while impaired; Weafer et al., 2008).

Consequently, the purpose of the present study was to examine the association between ADHD symptoms and subjective effects of alcohol, perceived intoxication, and alcohol craving. We hypothesized that ADHD symptoms would be positively associated with subjective effects of alcohol, perceived intoxication, and alcohol craving. The present study is a secondary data analysis of a larger parent study on impulsivity and alcohol use (Stamates et al., 2025). Participants in the parent study completed an in-person alcohol administration session and 10 days of ecological momentary assessment. Data presented in the current study are derived from the alcohol administration session only. Research questions and hypotheses were not pre-registered.

## 2. Materials and Methods

### 2.1. Participants and Procedures

A full description of participants and procedures is available in Stamates et al. (2025). To determine eligibility, participants completed a medical screening conducted by the research team. They were required to be between ages 21 and 30, own a smartphone, have consumed alcohol in the past month, have engaged in 1+ heavy drinking (4+/5+ drinks for females/males) episode(s) in the past six months, and have self-reported normal color vision. Participants were excluded if they had an Alcohol Use Disorder Identification Test (Saunders et al., 1993) score of 15+, a body mass index of 30+, any history of substance use treatment, psychiatric conditions, or medical contraindications (including breastfeeding). The final sample consisted of 26 participants (*M_age_* = 21.85, *SD* = 1.19; 61.5% female) who were mostly White (*n* = 22), 4th-year students (61.5%), lived in off-campus housing (*n* = 15), single (*n* = 25), employed part-time (*n* = 15), and did not affiliate with a fraternity or sorority (*n* = 21).

All participants were informed that they would receive an alcoholic beverage and provided written informed consent. The study was approved by the sponsoring institution’s Institutional Review Board (1515927-11). Participants were required to fast for two hours and abstain from alcohol for 24 h. At the start of the study, participants completed a breathalyzer test to verify a breath alcohol concentration [BrAC] of zero. Participants tested negative on urine drug and pregnancy (for females) tests. Participants were asked to bring a photo I.D. to verify they were of legal U.S. drinking age (21+). Participants then completed a battery of assessments.

After completing the assessments, participants were escorted to a simulated bar and were provided a 3:1 ratio of 3.94 mL/kg of Cherry 7-Up mixed with a 1.97 mL/kg dose of 40% alcohol/volume Smirnoff Red Label vodka and a splash of lime juice. This dose was selected so that BrACs reached approximately 0.08 g/210 L (Marczinski & Stamates, 2013) and was reduced to 87% for females so that there were no sex differences in BrACs. The dose was split across three glasses, and participants were instructed to finish each glass within five minutes, or 15 min in total. The exact content of the beverage was not disclosed to participants. BrAC readings were recorded at 30, 40, 60, 90, 120, 150, and 180 min. Participants were provided with a meal and stayed in the laboratory until BrACs reached 0.01 g/210 1. They received $50 for the alcohol administration session.

### 2.2. Measures

To assess ADHD symptoms, participants completed The Current Symptoms Scale (Barkley & Murphy, 2006), an 18-item self-report instrument that assessed the frequency of ADHD symptoms over the past 6 months and was based on a 4-point Likert scale of 0 (*Never or rarely*) to 3 (*Very often*). Higher total scores were indicative of greater ADHD symptom severity. For typical alcohol use, participants completed a Timeline Follow-Back (Sobell & Sobell, 1992), which measured the number of standard drinks consumed during the past 30 days. Participants were encouraged to access their phones to improve recall. The total number of drinks was summed. For subjective effects of alcohol, perceived intoxication, and alcohol craving, participants completed questionnaires at baseline and 30, 60, 90, 120, 150, and 180 min after administration. This included a 5-item visual analogue scale (Fillmore & Rush, 2001) by placing a tick mark on a 100 mm line from 0 = *Not at all* to 100 = *Very much* on items asking, “How much do you *feel* the drink right now?”, “How much do you *like* the drink right now?”, “How *intoxicated* or drunk do you feel right now?”, “How much do you *crave* to drink more alcohol right now?”, and “How willing are you to *drive* right now?”. All five scores at each timepoint were used in the present study. Participants also completed the Biphasic Alcohol Effects Scale (Martin et al., 1993) by rating seven items for stimulation (e.g., energized, excited) and for sedative effects (e.g., inactive, sluggish) on a 10-point scale from 1 = *Not at all* to 10 = *Extremely*. A score for *sedation* and *stimulation* was summed at each timepoint. For descriptive purposes, participants completed the Alcohol Use Disorder Identification Test (AUDIT; Saunders et al., 1993), a 10-item questionnaire, in which different aspects of drinking behavior during the last year are assessed. Total scores of eight or above are indicative of hazardous alcohol use. A total sum score was utilized, with higher scores indicating greater hazardous alcohol use.

### 2.3. Data Analytic Plan

Given that subjective response may vary on ascending and descending limbs of BrACs, we generated area under the curves (AUC) using GraphPad Prism (https://www.graphpad.com/, accessed on 27 April 2026, GraphPad Software, Inc., La Jolla, CA, USA; Dotmatics, 2023), which calculated each participant’s overall subjective response trajectory into a single index for each of the outcome variables (feel, like, willingness to drive, intoxication, craving, sedation, and stimulation). GraphPad Prism uses the trapezoidal rule to compute AUCs. Higher AUC values reflect greater subjective alcohol response over the study session. To examine the association between ADHD symptoms and subjective alcohol response, seven multiple regressions were examined to determine whether the total ADHD symptoms were associated with AUC scores. SPSS version 28 was used to test regressions.

## 3. Results

Descriptive statistics can be found in Table 1. Controlling for typical alcohol use, higher ADHD symptoms were associated with greater liking, willingness to drive, and craving. See Table 2 for regression model results.

## 4. Discussion

The results of the study revealed partial support for an association between ADHD symptoms and subjective effects of alcohol, perceived intoxication, and alcohol craving during the alcohol administration session. Specifically, we found that higher ADHD symptoms were positively associated with greater liking of the alcoholic beverage as well as craving or wanting to consume more alcohol. According to Robinson and Berridge (1993), “liking” a substance refers to the immediate pleasure gained from drinking alcohol and can be a precursor to craving. Robinson and Berridge (1993) also suggested that craving can result from hypersensitization from repeated substance use, resulting in neuroadaptation changes in the mesolimbic dopamine system. Results did not indicate that ADHD symptoms were related to other aspects of subjective alcohol response, such as stimulation or perceived intoxication, and thus, are somewhat inconsistent with Hendershot et al. (2015) and Weafer et al. (2008). Differential findings could be related to the age of the sample, as Hendershot et al. (2015) examined adults aged 18–21, or because individuals in our study did not have a history of ADHD diagnosis. Nonetheless, our findings are important because they suggest that individuals, specifically college students who reported higher ADHD symptoms, also tended to report subjective effects of alcohol, perceived intoxication, and alcohol craving that may reflect processes in the progression of addiction.

In the present study, individuals with greater ADHD symptoms also reported a greater willingness to drive after consuming alcohol despite reaching BrACs at the legal limit. Individuals with ADHD have been shown to experience greater impairment than those without ADHD when driving under the influence of alcohol (Weafer et al., 2008), and ADHD symptoms are associated with a greater likelihood of having an alcohol-involved motor-vehicle accident (Tinella et al., 2021, Tinella et al., 2024). Further, individuals with ADHD have been shown to take greater risks than the general population (Pollak et al., 2019, Pollak et al., 2023). Interventions may want to consider the association between ADHD and alcohol-related driving risk. Future research should explore reasons why individuals with elevated ADHD symptoms may be more willing to drive while intoxicated. In addition, willingness to drive may differ substantially in jurisdictions with lower legal limits (e.g., 0.00% or 0.02%) or in cultural contexts with different perceptions of driving while impaired. Therefore, the findings should be interpreted as reflecting perceived willingness to drive within the context of U.S. college students who were exposed to alcohol doses designed to approximate the U.S. legal threshold for driving.

There are limitations to the present study. First, generalizability of findings is limited due to a low sample size and the sample demographics (e.g., college students, mostly white). Second, analyses were underpowered to examine specific ADHD presentations based on symptom count (e.g., inattention vs. hyperactivity), which may be beneficial for clinical interventions. Third, while we were able to capture levels of subjective alcohol response across the three-hour assessment period, we were underpowered to examine specific subjective effects of alcohol (feel, like, sedation, and stimulation), perceived intoxication (intoxication and willingness to drive), and craving as BrACs rose and fell. Given that sensitivity to alcohol is dynamic (Treloar & Miranda, 2017), it is critical for future research to explore these associations among a larger sample size. Fourth, although individuals with a formal ADHD diagnosis were excluded, it is possible that some participants had undiagnosed ADHD. The present study is a secondary data analysis of a parent project focused on impulsivity, as measured by cognitive tasks, and alcohol use. Individuals with psychiatric conditions, including ADHD, were excluded to not confound performance on these tasks. However, young adults often have ADHD symptoms without a diagnosis (Blasé et al., 2009; Weyandt & DuPaul, 2008). Fifth, participants were tested individually, and thus, influence from peers was not assessed, which may limit generalizability. Fifth, willingness to drive was assessed within the context of a U.S. alcohol administration study in which participants reached BrACs near the legal driving limit of 0.08 g/210 L. Perceptions of driving fitness may be influenced by legal thresholds, cultural norms, and societal attitudes toward drinking and driving; therefore, willingness-to-drive ratings may not generalize to jurisdictions with different legal limits or cultural contexts. Lastly, we did not employ a placebo control. Hence, some of the effects may be, in part, due to alcohol expectancies.

In conclusion, higher ADHD symptoms were associated with greater “liking” and craving of the alcoholic beverage, and greater willingness to drive. Individuals with greater ADHD symptoms may be more likely to drink more alcohol during a drinking session. The present findings may also be useful when tailoring alcohol intervention and prevention efforts to help reduce risk for college students who report elevated ADHD symptomology.

## Figures and Tables

**Table 1:** Raw descriptive statistics of study variables.

	Baseline *M* (*SD*)	30 min. *M* (*SD*)	60 min. *M* (*SD*)	90 min. *M* (*SD*)	120 min. *M* (*SD*)	150 min. *M* (*SD*)	180 min. *M* (*SD*)
Alcohol Use	27.96 (20.88)	-	-	-	-	-	-
ADHD Symptoms	12.62 (6.77)	-	-	-	-	-	-
AUDIT	6.43 (2.79)	-	-	-	-	-	-
Feel	0	57.69 (26.96)	52.50 (20.80)	45.64 (28.57)	32.46 (19.65)	23.96 (19.12)	13.38 (10.85)
Like	0	59.92 (22.53)	52.50 (20.37)	41.40 (20.15)	30.88 (17.93)	28.58 (21.96)	19.46 (18.13)
Willingness to Drive	74.50 (39.86)	12.15 (14.41)	17.92 (25.13)	22.84 (29.42)	32.35 (32.02)	44.69 (33.80)	49.54 (38.05)
Intoxicated	0	53.77 (25.04)	48.63 (22.91)	41.72 (25.14)	32.69 (20.01)	22.38 (18.66)	11.00 (11.70)
Craving	7.60 (18.32)	38.38 (29.51)	33.71 (24.88)	22.84 (22.37)	13.77 (18.68)	10.54 (17.86)	7.54 (17.49)
Sedation	13.08 (5.99)	17.27 (8.03)	18.96 (9.68)	20.20 (11.54)	20.96 (11.37)	20.88 (11.47)	18.12 (11.14)
Stimulation	29.88 (13.54)	40.31 (14.39)	31.75 (14.72)	29.36 (15.68)	24.46 (13.13)	23.62 (13.17)	24.23 (13.84)

*Note*. *M* = mean, *SD* = standard deviation, AUDIT = Alcohol Use Disorder Identification Test.

**Table 2:** Regressions on ADHD symptoms associated with subjective alcohol response.

	B	β	SE	*p*	*R* ^2^
Criterion: Feel					0.05
ADHD symptoms	−1.45	−0.20	1.55	0.360	
Alcohol use	−0.16	−0.07	0.50	0.757	
Criterion: Like					0.19
ADHD symptoms	**2.87**	**0.45**	**1.23**	**0.029**	
Alcohol use	−0.35	−0.17	0.40	0.388	
Criterion: Willingness to Drive					0.35
ADHD symptoms	**5.51**	**0.53**	**1.79**	**0.005**	
Alcohol use	0.52	0.16	0.58	0.378	
Criterion: Intoxicated					0.12
ADHD symptoms	−2.05	−0.28	1.48	0.178	
Alcohol use	−0.34	−0.14	0.48	0.484	
Criterion: Craving					0.47
ADHD symptoms	**3.53**	**0.44**	**1.25**	**0.009**	
Alcohol use	**1.11**	**0.43**	**0.40**	**0.012**	
Criterion: Sedation					0.02
ADHD symptoms	0.23	0.06	0.81	0.776	
Alcohol use	0.12	0.10	0.26	0.640	
Criterion: Stimulation					0.10
ADHD symptoms	1.85	0.32	1.17	0.127	
Alcohol use	−0.08	−0.04	0.38	0.832	

Note. Significant effects are bolded. B = unstandardized estimates for path; β = standardized estimates for path; SE = standard error.

## Data Availability

Data may be made available upon request.

## Abbreviations

The following abbreviations are used in this manuscript:

ADHD	Alcohol use and attention-deficit/hyperactivity disorder
AUC	Area under the curve
AUD	Alcohol use disorder
AUDIT	Alcohol use disorder identification test
BrAC	Breath alcohol concentration
